# Inhibition of the phosphoinositide 3-kinase pathway decreases innate resistance to lipopolysaccharide toxicity in TLR4 deficient mice

**DOI:** 10.1186/1423-0127-21-20

**Published:** 2014-03-11

**Authors:** Johnson Chia-Shen Yang, Shao-Chun Wu, Cheng-Shyuan Rau, Tsu-Hsiang Lu, Yi-Chan Wu, Yi-Chun Chen, Ming-Wei Lin, Siou-Ling Tzeng, Chia-Jung Wu, Ching-Hua Hsieh

**Affiliations:** 1 Department of Plastic and Reconstructive Surgery, Kaohsiung Chang Gung Memorial Hospital and Chang Gung University College of Medicine, No.123, Ta-Pei Road, Niao-Sung District, Kaohsiung City 833, Taiwan; 2 Department of Anesthesiology, Kaohsiung Chang Gung Memorial Hospital and Chang Gung University College of Medicine, Kaohsiung City 833, Taiwan; 3 Department of Neurosurgery, Kaohsiung Chang Gung Memorial Hospital and Chang Gung University College of Medicine, Kaohsiung City 833, Taiwan

**Keywords:** Lipopolysaccharide (LPS), Toll-like receptor 4 (TLR4), Phosphoinositide 3-kinase (PI3K)

## Abstract

**Background:**

Upon lipopolysaccharide (LPS) stimulation, activation of both the Toll-like receptor 4 (TLR4) and phosphoinositide 3-kinase (PI3K) pathways serves to balance proinflammatory and anti-inflammatory responses. Although the antagonist to TLR4 represents an emerging promising target for the treatment of sepsis; however, the role of the PI3K pathway under TLR4-null conditions is not well understood. This goal of this study was to investigate the effect of inhibition of PI3K on innate resistance to LPS toxicity in a murine model.

**Results:**

The overall survival of the cohorts receiving intraperitoneal injections of 100, 500, or 1000 μg LPS from *Escherichia coli* serotype 026:B6 after 7 d was 100%, 10%, and 10%, respectively. In contrast, no mortality was noted after 500-μg LPS injection in *Tlr4*^-/-^ mice. When the PI3K inhibitor LY294002 was injected (1 mg/25 g body weight) 1 h prior to the administration of LPS, the overall survival of the *Tlr4*^-/-^ mice was 30%. In the *Tlr4*^-/-^ mice, the LPS injection induced no NF-κB activation but an increased Akt phosphorylation in the lung and liver, when compared to that of the C57BL/6 mice. Injection of 500 μg LPS led to a significant induction in O_2_^-^ detected by electron paramagnetic resonance (EPR) spin trapping spectroscopy in the lung and liver at 3 and 6 h in C57BL/6 but not *Tlr4*^-/-^ mice. Addition of LY294002 only significantly increased the O_2_^-^ level in the lung and liver of the *Tlr4*^-/-^ mice but not in the C57BL/6 mice following 500-μg LPS injection. In addition, the serum IL-1β and IL-2 levels were more elevated in C57BL/6 mice than in *Tlr4*^-/-^ mice. Notably, IL-1β and IL-2 were significantly increased in *Tlr4*^-/-^ mice but not in the C57BL/6 mice when the PI3K pathway was inhibited by LY294002 prior to LPS injection.

**Conclusions:**

In this study, we demonstrate that innate resistance to LPS toxicity in *Tlr4*^-/-^ mice is impaired by inhibition of the PI3K pathway, with a corresponding increase in mortality and production of tissue O_2_^-^ and inflammatory cytokines.

## Background

Lipopolysaccharide (LPS) from gram-negative bacteria is a potent Toll-like receptor 4 (TLR4) ligand and an inflammatory mediator that activates well characterized pathways involving nuclear factor-kappa B (NF-κB) signal transduction and subsequent expression of inflammatory cytokines [1]. TLRs signal through two main pathways: a myeloid differentiation factor (MyD)88-dependent pathway that acts via NF-κB to induce pro-inflammatory cytokines, and a MyD88-independent pathway that acts via type I interferons to increase the expression of interferon-inducible genes [2,3]. TLR4 stimulation also induces concomitant activation of the phosphoinositide 3-kinase (PI3K) and the downstream target serine/threonine kinase Akt [4-9]. The p85 regulatory domain subunit of PI3K has been reported to associate with TLR4 in cells, and co-immunoprecipitation experiments demonstrated that MyD88 binds p85 upon LPS stimulation [10,11]. It has been demonstrated that, upon LPS stimulation, the kinetics of MyD88 and PI3K recruitment to TLR4 are similar [11]. Moreover, MyD88 and PI3K constitutively bind as a dimer and have a direct role in the activation of PI3K [12,13]. The induced concomitant activation of the PI3K/Akt pathway following TLR4 stimulation was recently shown to negatively regulate the pro-inflammatory response both *in vitro* and *in vivo*[4-9]. LPS-induced phosphorylation of Akt was reported to be completely TLR4-dependent [14], and can be suppressed by a specific PI3K inhibitor, LY294002 [15].

The PI3K/Akt signaling pathway is one of the most critical pathways involved in regulation of cell survival [16] and inflammatory responses [17]. The PI3K/Akt signaling pathway may be an endogenous negative feedback regulator and/or compensatory mechanism that serves to limit pro-inflammatory and apoptotic events in response to injurious stimuli [18-20]. Recent evidence indicates that there is crosstalk between the TLR and PI3K/Akt signaling pathways [11,12,18-20]. LPS-induced TLR4 stimulation protects monocytes and human dendritic cells from apoptosis through PI3K/Akt- and NF-κB-dependent mechanisms by regulating the phosphorylation of NF-κB p65 at serine 536 and Akt at serine 473 [12]. In addition, in a murine model of cecal ligation and puncture-induced polymicrobial sepsis, inhibition of PI3K activity increased serum cytokine levels and mortality [20]. In contrast to these findings, stimulation of the PI3K pathway was correlated with improved outcome [20]. Administration of endotoxin to PI3Kγ-knockout mice resulted in decreased acute lung injury, suggesting that the PI3K pathway plays an important role in the pathophysiology of endotoxic injury [21].

Sepsis mice induced by the administration of LPS were employed as a redox disruption model because sepsis is well known to be a systemic inflammatory response syndrome related to the generation of reactive oxygen species (ROS) [22]. LPS has been shown to generate ROS through the stimulation of LPS-binding proteins and Toll-like receptors [23]. Furthermore, the released cytokines were shown to induce ROS generation in endothelial cells [24]. The expression level of TLR4 determines the degree of LPS susceptibility in mice [25]. Manipulation of TLR4 activation has been hypothesized to modulate the innate resistance to LPS toxicity. For example, C57BL/10ScCr mice carry a deletion of the *Tlr4* gene, and are thus refractory to LPS effects [25,26]. TLR4 antagonism also restores the function of septic organs during endotoxemia [27,28]. The simultaneous activation of both the TLR4 and PI3K pathways upon LPS stimulation has been reported to balance the pro-inflammatory and anti-inflammatory response [29]; Although the antagonist to TLR4 represents an emerging promising target for the treatment of sepsis; however, the role of PI3K pathway under TLR4-null conditions is not well understood. In this study, we demonstrate that innate resistance to LPS toxicity in *Tlr4*^-/-^ mice is reduced by the inhibition of the PI3K pathway, with a corresponding increase in mortality and production of tissue superoxide and inflammatory cytokines.

## Methods

### Animals

Eight to twelve week old male mice weighing 25–30 g were used in the study. *Tlr4*^*-/-*^ (C57BL/10ScNJ) mice were purchased from Jackson Laboratory (Bar Harbor, ME, USA). C57BL/6 mice were purchased from the National Laboratory Animal Center, Taiwan. The murine strain C57BL/10ScNJ mice have a deletion of the *Tlr4* gene that results in absence of both mRNA and protein and thus in defective response to LPS stimulation. C57BL/6 was used as a control. All housing conditions and surgical procedures, analgesia, and assessments were in accordance with national and institutional guidelines, and an Association for Assessment and Accreditation of Laboratory Animal Care (AAALAC)–accredited SPF facility was used. The animal protocols were approved by the Institutional Animal Care and Use Committee (IACUC) of Kaohsiung Chang Gung Memorial Hospital.

### Survival studies

LPS from *Escherichia coli* serotype 026:B6 (catalog no. L3755) was purchased from Sigma-Aldrich (St. Louis, MO, USA). To profile the LPS toxicity, C57BL/6 mice were injected intraperitoneally (i.p.) with 100, 500, 1000 μg of LPS reconstituted in 100 μL of phosphate-buffered saline (PBS) (n = 10 in each group) for survival studies. The mice were returned to their cages after LPS injection and closely monitored for up to 7 d. Mice were given *ad libitum* access to food and water at all times. Additional groups of *Tlr4*^-/-^ mice received i.p. injections of 500 μg LPS in the presence or absence of i.p. PI3K inhibitor LY294002 (1 mg/25 g body weight, Sigma-Aldrich) and were observed for survival studies (n = 10 in each group). LY294002 was injected 1 h prior to the administration of LPS.

### Blood and tissue samples

C57BL/6 and *Tlr4*^-/-^ mice were injected i.p. with 500 μg LPS and sacrificed at 3- or 6-h post-injection. The control group was injected with 100 μL PBS. Additional groups of *Tlr4*^-/-^ mice received i.p. injections of (1 mg/25 g body weight) LY294002 1 h prior to the injection of 500 μg LPS (n = 6 in each group). Whole blood was drawn from the mice, and tissues, including the lungs, liver, and spleen, were harvested for western blot analysis of Akt phosphorylation and nuclear translocation of p65 as well as the measurement of superoxide (O_2_^-^) production. Whole blood samples (1 mL per mouse) were collected into tubes containing an anticoagulant and incubated at room temperature for 15 min prior to centrifugation at 3,000 × g for 10 min. White blood cells (WBCs) were slowly removed from the corresponding layers and the serum was extracted and stored at -80°C before processing for cytokine assays.

### Western blot analysis

The harvested lung, liver, and spleen tissues from wild type C57BL/6 mice and *Tlr4*^-/-^ mice injected with 500 μg LPS in the presence or absence of LY294002 were harvested for detection of pAkt/Akt and nuclear p65/lamin B1 proteins. We extracted the cytoplasmic and nuclear protein fractions using NE-PER extraction reagents according to the manufacturer’s protocol (Pierce Biotechnology, IL, USA). Cytoplasmic and nuclear protein extracts were used for Western blot analysis. The protein samples (30 μg) were resolved on a 10% SDS-polyacrylamide gel and transferred to polyvinylidenedifluoride membranes. The membranes were then blocked with 5% skim milk in Tween-20/ PBS and incubated with various primary antibodies, including rabbit anti–phospho-Akt (Ser473), anti-Akt (Cell Signaling Technology, Danvers, MA, USA), anti-p65, and anti-lamin B1 (Santa Cruz, CA, USA) at 4°C overnight. The blots were then incubated with horseradish peroxidase–conjugated secondary antibodies at room temperature for 60 min, and developed using the ECL™ Western Blotting System (Amersham Pharmacia Biotech, Aylesbury, UK). The protein bands were quantified with the FluorChem 8900 imaging system and the AlphaEaseFC software (Alpha Innotech Corp, CA, USA).

### Superoxide measurement

Superoxide formation was measured using electron paramagnetic resonance (EPR) spin trapping spectroscopy [30]. Briefly, harvested lung, liver, and spleen tissues were homogenized with 10 μg/mL chelex-treated PBS containing aprotinin, 0.5 μg/mL leupeptin, 0.7 μg/mL pepstatin, and 500 μM PMSF. The protein samples (30 μg) were mixed with 1 mM 1-hydroxy-3-carboxypyrrolidine (CPH) and 0.1 mM diethyl-tetrapentaacetic acid (DTPA) to chelate the ions of transition metals. The mixture was loaded in 50-μL glass capillary tubes (Wilmad Glass, Buena, NJ, USA). The electron paramagnetic resonance spectra were recorded using an EMX Plus EPR spectrometer (Bruker Biospin, Rheinstetten, Germany) equipped with an EMX-m40X microwave bridge operating at 9.88 GHz.

### Cytokine assays

The concentrations of IL-1β and IL-2 in the serum samples were determined using enzyme-linked immunosorbent assay (ELISA) kits for IL-1β and IL-2 (lnvitrogen Corporation, CA, USA), as per the manufacturer’s protocol. Sample concentrations were then calculated from a standard curve. Results are expressed as picograms per milligram (pg/mg) of protein.

### Statistical analysis

All results are presented as the mean ± standard error. An overall analysis of the differences between the group means was calculated by a one way analysis of variance (ANOVA) and an appropriate post hoc test using SPSS statistical software (SPSS 18, Chicago, IL, USA). A value of P < 0.05 was considered to indicate statistical significance.

## Results

**Figure 1:**
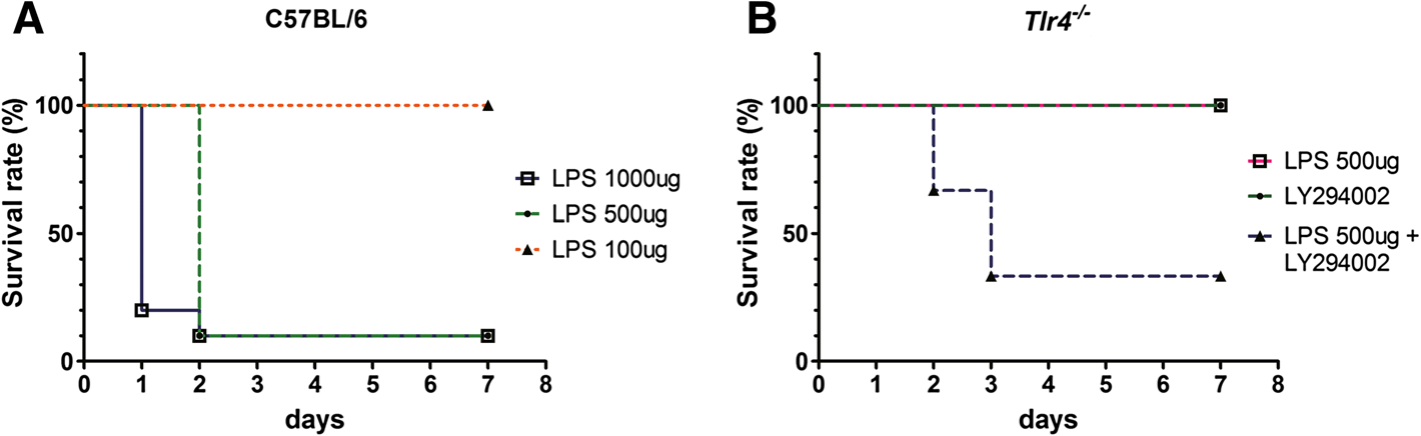
**Survival study. (A)** Survival rate of the C57BL/6 mice receiving intraperitoneal injections of 100, 500, or 1000 μg LPS from *Escherichia coli* serotype 026:B6. Mice were monitored up to 7 days (n = 10 in each group) **(B)** Survival rate of the *Tlr4*^-/-^ mice receiving intraperitoneal injection of LY294002 (1 mg/25 g body weight) alone, 500 μg LPS alone, or 500 μg LPS with LY294002 injected 1 h prior.

Immediately after LPS injection, the mice showed pronounced symptoms, including reduced mobility, ruffled fur, hunched appearance, and lethargy. As shown in Figure 1, after injection of 1000-μg LPS, 80% of the C57BL/6 mice died within the first 24 h and 90% of the mice died within 48 h. After injection of 500 μg LPS, no mice died within the first 24 h; however, 90% of the mice died within 48 h. No mortality was noted throughout the experiment after injection of 100 μg of LPS. The overall survival after 7 d in the mouse groups receiving 100-, 500-, and 1000-μg LPS injections was 100%, 10%, and 10%, respectively (Figure 1A). In contrast, after injection of 500 μg LPS in the *Tlr4*^-/-^ mice, no mortality was noted throughout the experiment. In addition, the symptoms observed following LPS injection were less pronounced during the entire observation period and the mice regained full mobility quickly. When 1 mg/25 g body weight LY294002 was injected 1 h prior to the administration of LPS, 30% and 70% of the *Tlr4*^-/-^ mice died within 48 and 72 h, respectively (Figure 1B). The overall 7-d survival of the *Tlr4*^-/-^ mice following PI3K inhibition and injection of 500 μg LPS was 30%. Injection with 1 mg/25 g body weight LY294002 alone did not cause mortality in the *Tlr4*^-/-^ mice.

**Figure 2:**
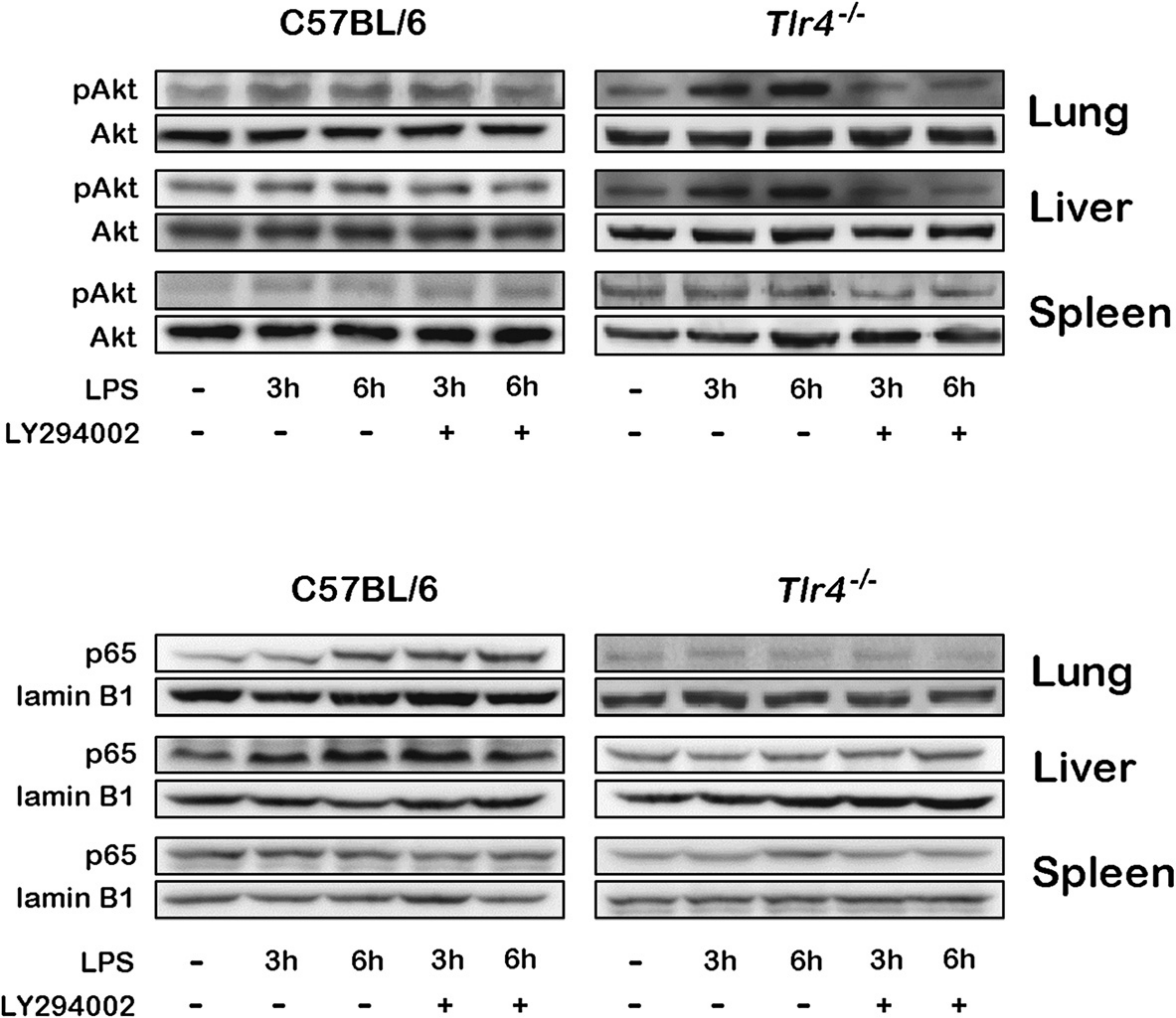
**Akt phosphorylation and p65 nuclear translocatoin in the lung, liver, and spleen tissues of C57BL/6 and *Tlr4***^**-/- **^**mice at 3 and 6 h after intraperitoneal injection of 500 μg LPS in the absence or presence of LY294002 injected 1 h prior in quadruplicate.**

**Figure 3:**
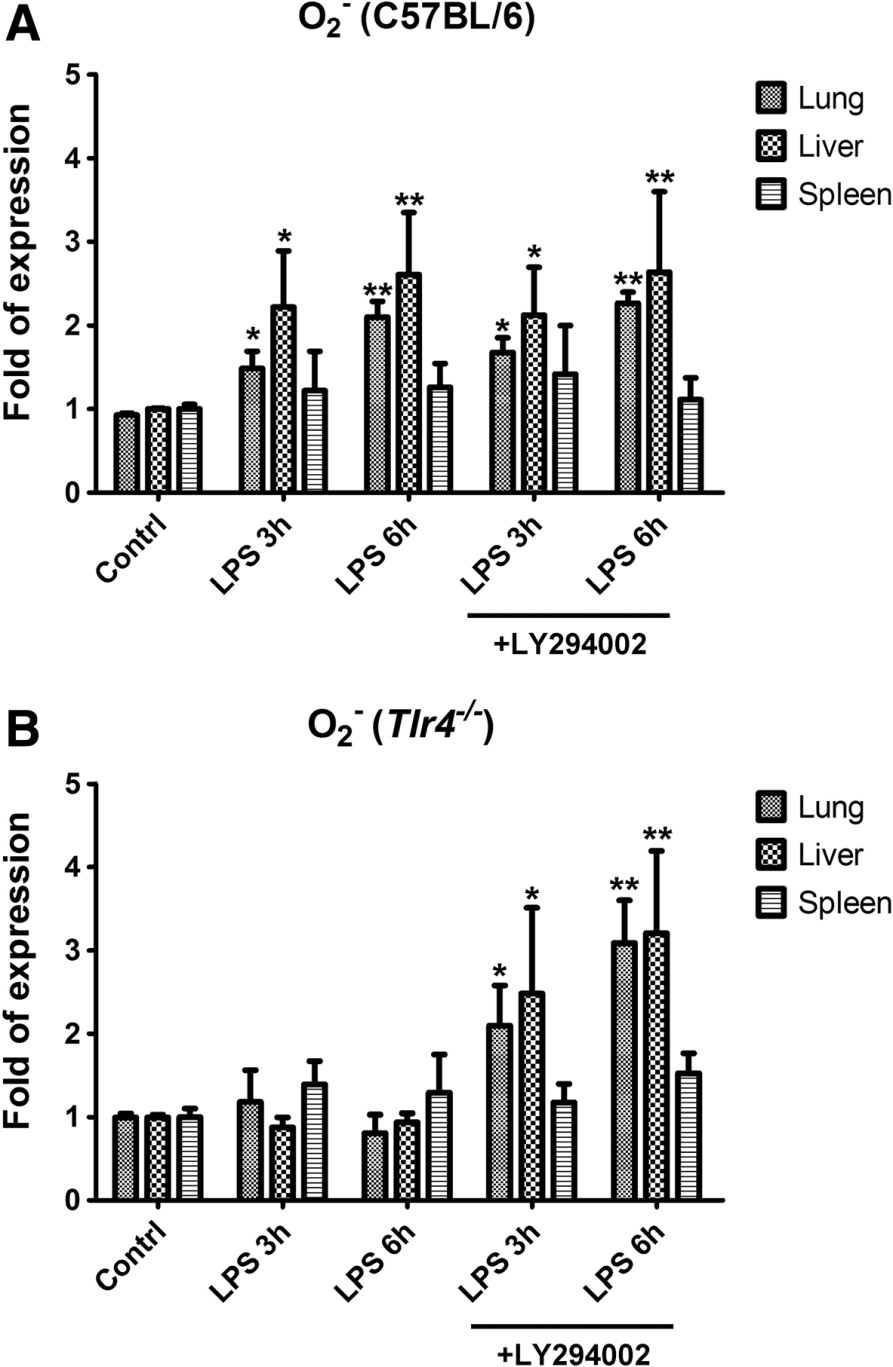
**Expression of superoxide.** O_2_^-^ level detected by electron paramagnetic resonance (EPR) spin trapping spectroscopy in the lung, liver, and spleen tissues of the **(A)** C57BL/6 or **(B)***Tlr4*^-/-^ mice at 3 and 6 h after intraperitoneal injection of 500 μg LPS in the absence or presence of LY294002 injected 1 h prior. Bars represent mean ± SEM values from 6 experiments; *P < 0.05, **P < 0.01 vs. control.

Injection of 500 μg LPS in the C57BL/6 mice induced activation of Akt at 3 and 6 h in the lung and liver (Figure 2); however, injection of 500 μg LPS in the *Tlr4*^-/-^ mice induced more Akt phosphorylation after 3 and 6 h in the lung and liver. There was no Akt phosphorylatoin being detected in the spleen tissue of either C57BL/6 or *Tlr4*^-/-^ mice following LPS injection. Addition of LY294002 one hour prior to LPS injection effectively blocked the activation of Akt in the lung and liver of the *Tlr4*^-/-^ mice at 3 and 6 h post-treatment. In contrast, injection of 500 μg LPS in the C57BL/6 mice induce the signficant NF-κB activation that presented as p65 nuclear translocation at 6 h in the lung, at 3 h and 6 h in the liver, but not in the spleen tissue. Addition of LY294002 one hour prior to LPS injection did not inhibit the p65 nuclear translocation in the lung and liver of the C57BL/6 mice at 3 and 6 h post-treatment. In the *Tlr4*^-/-^ mice, LPS injection did not induce significant p65 nuclear translocation in all three tissues regardless of the presence or absence of LY294002 pretreatment. Injection of 500 μg LPS resulted in significantly elevated EPR signals, which were indicative of elevated O_2_^-^ levels, in the lung and liver of the C57BL/6 mice at 3 and 6 h post-treatment (Figure 3A). Addition of LY294002 did not significantly change the elevated O_2_^-^ levels in the lung and liver of the C57BL/6 mice at 3 and 6 h post-treatment. No change in the EPR signals was noted in the spleen tissue after LPS injection at either 3 or 6 h. In contrast, no significant elevation in EPR signals was found in the lung, liver, or spleen of *Tlr4*^-/-^ mice receiving injection of 500 μg LPS (Figure 3B). Inhibition of the PI3K pathway with LY294002 one hour prior to LPS injection significantly elevated the EPR signals in the lung and liver tissue of the *Tlr4*^-/-^ mice administered 500 μg LPS. LY294002 pretreatment alone induced no remarkable change in EPR signals in the spleen (Figure 3B).

**Figure 4:**
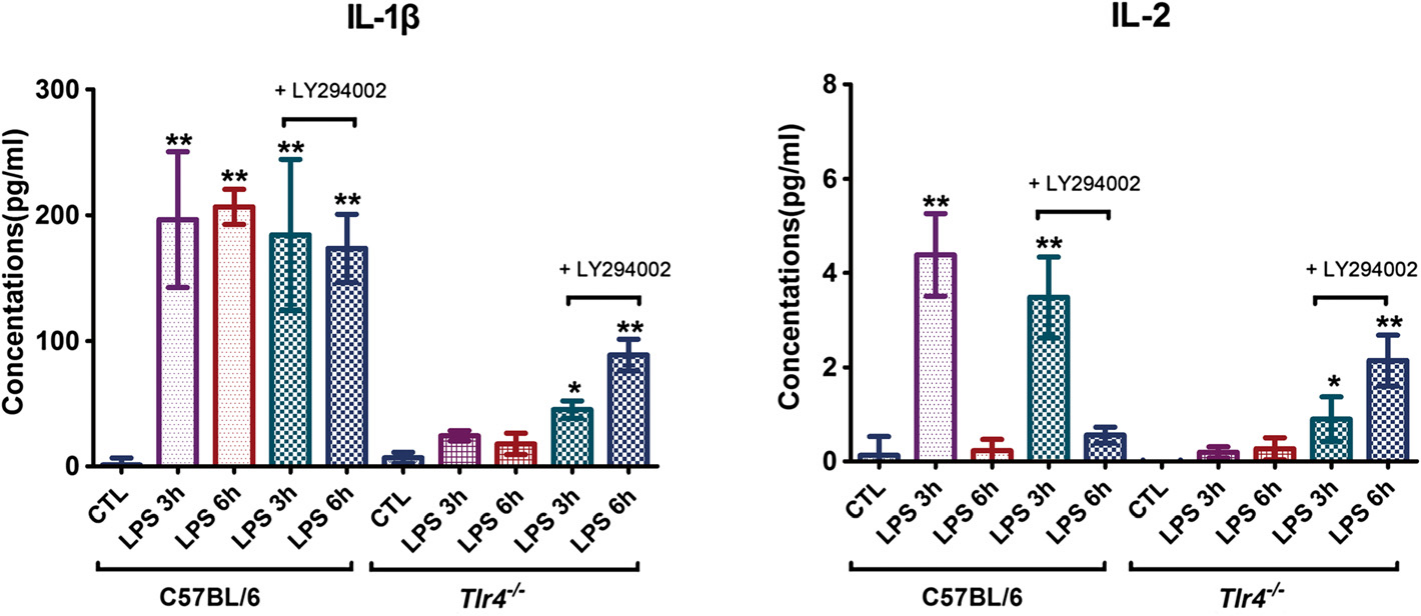
**Cytokine expression.** Levels of IL-1β and IL-2 in the serum of C57BL/6 or *Tlr4*^-/-^ mice at 3 and 6 h after intraperitoneal injection of 500 μg LPS in the absence or presence of LY294002 injection 1 h prior. CTL: control. Bars represent mean ± SEM values of 6 experiments; *P < 0.05, **P < 0.01 vs. control.

As shown in the Figure 4, 500-μg LPS injections in the C57BL/6 mice significantly induced the expression of IL-1β at 3 and 6 h as well as IL-2 at 3 h in the serum post-treatment. LY294002 administration did not significantly change the expression of IL-1β and IL-2 in the serum of C57BL/6 mice 3 and 6 h after injection of 500 μg LPS. In contrast, injection of 500 μg LPS in the *Tlr4*^-/-^ mice resulted in a significantly lower induction of IL-1β expression at 3 and 6 h as well as decreased IL-2 levels 3 h post-treatment, as compared to that in C57BL/6 mice. LY294002 administration significantly increased the expression of IL-1β and IL-2 in the serum of *Tlr4*^-/-^ mice 3 and 6 h after injection of 500 μg LPS. However, in the *Tlr4*^-/-^ mice, the increase in serum IL-1β following PI3K pathway inhibition and LPS stimulation was not as high as that in similarly treated C57BL/6 mice. The increase in serum IL-2 levels in *Tlr4*^-/-^ mice receiving LY294002 was also not as high as that in C57BL/6 mice 3 h post-LPS treatment. However, at 6 h post-treatment, the serum IL-2 levels in *Tlr4*-/- mice receiving LY294002 with LPS stimulation were significantly higher than that in C57BL/6 mice under the same treatment conditions.

## Discussion

In this study, we demonstrate that the innate resistance to LPS toxicity in *Tlr4*^-/-^ mice is reduced by inhibition of the PI3K pathway, with a subsequent increase in mortality and production of tissue superoxide and inflammatory cytokines. There were many evidences that the the PI3K pathway served as up- and down-stream relationship to the TLR4 signaling pathway [4-9]. However, there were some reports describing a parallel function of PI3K pathway to balance the effect of TLR4 activation [18-20]. In this study, LPS injection induced no NF-κB activation but an increased Akt phosphorylation in the lung and liver of the *Tlr4*^-/-^ mice, when compared to that of the C57BL/6 mice, implying there is an important role of PI3K pathway in mediating the inflammatory response in the TLR4-null condition. These results also suggest that, following LPS stimulation, there may be an alternative pathway for the activation of PI3K that is independent of the TLR4/MyD88 axis, and that this PI3K pathway upon activation exerts anti-inflammatory effects under TLR4-null conditions. Previously, it has also been demonstrated that inhibition of PI3K activity increases LPS-induced interferon β (IFN-β) synthesis [31,32]. In contrast, the LPS-induced phosphorylation of Akt is markedly diminished in macrophages from IFN-β^-/-^ mice [33]. However, relatively little is known regarding the contribution of the MyD88-independent pathway to PI3K activation.

TLR4 activates immune responses by sensing not only microbial structures such as bacterial LPS but also some endogenous “danger” molecules released by damaged host cells [34,35]. Inflammatory responses induced by sterile stimuli are very similar to responses during infection, and are mediated by common receptors and pathways [36,37]. Multiple studies using TLR^-/-^ animals or targeted gene silencing of *Tlr4* have shown that both inflammation and injury responses like those in ischaemia/reperfusion (I/R) are partially TLR4-dependent [14,38-40]. Similarly, there is a balance between the TLR/NF-κB and PI3K/Akt signaling pathways in mediating the inflammation and injury response during sterile inflammation. For example, activation of the PI3K/Akt signaling pathway has been reported to be associated with decreased myocardial ischemic injury through the modulation of TLR4-mediated signaling [41]. The ability of LPS pre-treatment to induce cardioprotection following ischemia/reperfusion is mediated through a PI3K/Akt-dependent mechanism [12]. Pharmacological inhibition of PI3K with LY294002 abrogated the protective effect of LPS pre-treatment in myocardial I/R injury [12].

In a study of time courses expression of inflammatory mediators, including IL-1α, IL-1β, IL-2, IL-3, IL-4, IL-5, IL-6, IL-9, IL-12(p40), IL-13, Eotaxin (CCL11), G-CSF, GM-CSF, IFN-γ, KC (CXCL1), MCP-1 (CCL2), MIP-1α, (CCL3), MIP-1β (CCL4), RANTES (CCL5) and TNF-α, of C57BL/6 mice receiving intraperitoneal injection of LPS [42], significant inductions of all mediators were found, with most mediators reached their maximum around 6–12 h. Interesting, there was a rapid fall following only 1 h rapid surge of TNFα. In addition, although there was extraordinary high amounts of IL-6, which was deemed as prototypical cytokine for endotoxemia and sepsis studies, around 3–12 h, there was no statistically significant change at 24 h. In this study, we chosed IL-1β and IL-2 as representive cytokines for measurement and revealed that the administration of LY294002 prior to LPS injection significantly increased the serum expression of IL-1β and IL-2 in *Tlr4*^-/-^ mice 3 and 6 h after injection of 500 μg LPS. These data are consistent with a report that inhibition of PI3K *in vivo* resulted in significant increases in circulating IL-1β, IL-2, IL-6, IL-10, IL-12, and TNF-α during polymicrobial sepsis [20] as well as *in vitro* studies [6] demonstrating the inhibitory effect of the PI3K/Akt pathway on release of these cytokines. Notably, in the absence of LPS stimulation, inhibitors of Akt or PI3K had no discernible effect on pro- or anti-inflammatory cytokine production as compared to untreated controls [43]. Our results demonstrated that, in the *Tlr4*^-/-^ mice, the increase in serum IL-1β and IL-2 levels 3 h after LPS treatment and a concurrent inhibition of the PI3K pathway was still not as high as the corresponding increase in serum cytokine levels in C57BL/6 mice. In addition, at 6 h, the level of IL-2 in *Tlr4*^-/-^ mice receiving LY294002 was significantly higher than that in the serum of the C57BL/6 mice after LPS treatment. The significance and mechanism of this differential regulation of *in vivo* cytokine expression after PI3K inhibition in the *Tlr4*^-/-^ mice is not clear. However, it should also be noted that the precise role of circulating cytokines in the pathophysiology of sepsis/septic shock is still controversial [44], and there is no definitive cause-and-effect relationship between systemic cytokine levels and survival outcome in sepsis [44].

## Conclusion

In this study, we demonstrate that innate resistance to LPS toxicity in *Tlr4*^*-/-*^ mice is impaired by inhibition of the PI3K pathway, with a corresponding increase in mortality and production of tissue O_2_^-^ and inflammatory cytokines.

## Competing interests

The authors declare that they have no competing interests.

## Authors’ contributions

JCY, SCW, and CSR contributed to analysis and acquisition of all data and the writing of the manuscript. THL and SLT participated in the animal surgery and acquisition of the study specimens. YCC and CJW participated in Western blotting experiment and superoxide measurement. YCW contributed to ELISA study. MWL participated in analysis of all data. CHH contributed to the design of animal study, interpretation of the analyzed results and the writing of the manuscript. All authors read and approved the final manuscript.
